# Letrozole-associated controlled ovarian hyperstimulation in breast cancer patients versus conventional controlled ovarian hyperstimulation in infertile patients: assessment of oocyte quality related biomarkers

**DOI:** 10.1186/s12958-018-0443-x

**Published:** 2019-01-03

**Authors:** Oranite Goldrat, Geraldine Van Den Steen, Eric Gonzalez-Merino, Julie Dechène, Christine Gervy, Anne Delbaere, Fabienne Devreker, Viviane De Maertelaer, Isabelle Demeestere

**Affiliations:** 1 Fertility Clinic, Department of Obstetrics and Gynecology, CUB-Hôpital Erasme, Université Libre de Bruxelles (ULB), Route de Lennik 808, Brussels, Belgium; 2 Research Laboratory on Human Reproduction, Université Libre de Bruxelles (ULB), Route de Lennik 808, Brussels, Belgium; 3 Laboratory of Chemistry, CUB-Hôpital Erasme, Université Libre de Bruxelles (ULB), Route de Lennik 808, Brussels, Belgium; 4 IRIBHM and SBIM, Université Libre de Bruxelles (ULB), Route de Lennik 808, Brussels, Belgium

**Keywords:** Breast cancer, Controlled ovarian hyperstimulation, Letrozole, Oocyte quality, Follicular fluid, Cumulus cells, Gene expression

## Abstract

**Background:**

Fertility preservation (FP) protocols in case of breast cancer (BC) include mature oocyte cryopreservation following letrozole associated controlled ovarian hyperstimulation (Let-COH). To date, the impact of Let-COH on the follicular microenvironment has been poorly investigated, although a high androgen/estrogen ratio was previously associated with low oocyte quality.

**Methods:**

In this prospective study, follicular fluid (FF) steroid levels (estradiol, testosterone, progesterone) and cumulus cell (CC) gene expression related to oocyte quality (*HAS2, PTGS2, GREM1*) were compared between 23 BC patients undergoing Let-COH for FP and 24 infertile patients undergoing conventional COH without letrozole. All patients underwent an antagonist COH cycle, and ovulation was triggered with hCG or GnRHa in both groups.

**Results:**

FF estradiol levels were significantly lower while testosterone levels were significantly higher in the study group compared to controls irrespective of the trigger method. However, estradiol levels increased significantly with GnRHa triggering compared to hCG in the study group (median = 194.5 (95.4–438) vs 64.4 (43.8–152.4) ng/ml, respectively, *p* < 0.001), but not in the control group (median = 335.5 (177.5–466.7) vs 354 (179–511) ng/ml, respectively). After hCG trigger, Cumulus cell (CC) gene expression was lower in the study group compared to the control group, and difference was significant for *PTGS2*. Conversely, CC gene expression of *PTGS2* and *GREM1* was significantly higher in the study group compared to controls when ovulation was triggered with GnRHa.

**Conclusions:**

Let-COH triggered with hCG may negatively impact oocyte quality. However, ovulation triggering with GnRHa may improve the oocyte microenvironment and cumulus cell genes expression in Let-COH, suggesting a positive impact on oocyte quality in breast cancer patients.

**Trial registration:**

Clinicaltrials.gov -NCT02661932, registered 25 January 2016, retrospectively registered.

**Electronic supplementary material:**

The online version of this article (10.1186/s12958-018-0443-x) contains supplementary material, which is available to authorized users.

## Background

Recent advances in primary systemic therapy have greatly improved relapse-free survival of young breast cancer (BC) patients [1, 2], and increased survival has led clinicians to focus on long-term quality of life issues such as access to motherhood. Moreover, pregnancy after BC treatment does not increase relapse risk and may even improve overall survival [3]. Nevertheless, chemotherapy including alkylating agents may cause infertility or premature ovarian failure, reducing patient’s chances to conceive [4]. Furthermore, in patients with hormone-sensitive disease, endocrine therapy is administered for several years. Consequently, aging should also be taken into consideration as a crucial fertility decline factor [5–7]. Fertility preservation (FP) has hence become a priority for patients requiring chemotherapy. Oocyte and/or embryo vitrification before treatments are usually offered to BC patients [8]. However, high estradiol level observed during controlled ovarian hyperstimulation (COH) before oocyte collection has been subject of debate regarding possible proliferative effects on the tumor. Consequently, more than a decade ago, a new COH protocol associated with letrozole, a type II nonsteroidal competitive aromatase inhibitor, was developed (Let-COH) to collect several mature oocytes while potentially avoiding negative effects of estrogens on tumor growth [9, 10]. Through its competitive action on the aromatase enzyme [11, 12], letrozole prevents the aromatization of androgens to estrogens, which may induce significant changes in the endocrine follicular environment and impact oocyte competence. However, an estrogenic environment was previously associated with better oocyte outcomes and anti-atretic effect, while elevated androgen/estrogen ratio was reported to induce granulosa cell apoptosis, associated with low quality or degenerating oocytes [13–17]. Recent studies have shown significantly improved oocyte yield in BC patients undergoing Let-COH for FP compared to conventional COH for elective oocyte cryopreservation, as well as in infertile patients undergoing IVF with Let-COH or conventional COH [18, 19].

Nevertheless, as developmental competence of frozen oocytes/embryos of large cohorts of BC patients might be known only in several years through live-birth rates, the assessment of indirect markers in the microenvironment surrounding the oocyte is an attractive approach to evaluate the oocyte quality. Several genes expressed in cumulus cells (CC) have been evaluated as potential markers of oocyte competence, offering an attractive approach to select embryos with the highest developmental potential. Among them, Hyaluronic acid synthase 2 (*HAS2*), Prostaglandin-endoperoxide synthase-2 (*PTGS2*) and Gremlin1 (*GREM1*) have been associated with higher oocyte competence and good embryo quality, given their interaction with oocyte secreted factors and their role in CC expansion during oocyte maturation. Although not always statistically significant, a higher expression of these genes has been associated with higher oocyte competence [20–25].

The impact of Let-COH for FP in BC patients on the follicular milieu remains poorly investigated so far. The objective of this study was hence to compare the impact on follicular fluid (FF) steroid levels and CC gene expression of the Let-COH protocol for FP in BC patients and conventional COH in infertile patients.

## Materials and methods

### Population

BC patients were enrolled in the BROVALE trial, a prospective study conducted between December 2012 and February 2017. The study group included 23 young BC patients of 18–41 years with non-metastatic disease and basal follicle stimulating hormone (FSH) < 20 IU/L, undergoing oocyte/embryo freezing for FP with Let-COH protocol. The control group included 24 infertile women aged < 41 years, treated with first or second ICSI cycles for tubal, male, and/or idiopathic infertility, undergoing similar ovarian stimulation for intra-cytoplasmic sperm injection (ICSI), without letrozole (conventional COH). Patients with severe endometriosis, ovarian insufficiency or severe polycystic ovary syndrome (PCOS), based on Anti-Müllerian Hormone (AMH) levels < 0.5 or > 8 ng/ml respectively, were excluded from this analysis.

### Controlled ovarian hyperstimulation protocols

For this study, patients in both groups underwent a gonadotropin releasing hormone (GnRH) antagonist cycle (Cetrorelix, Cetrotide® 0.25 mg, Serono, Germany) with recombinant FSH (rFSH 150–300 IU/day, Gonal-f®, Serono, Germany) and were triggered using 10,000 IU hCG (Pregnyl®, MSD, Switzerland) or 0.2 mg Triptorelin (GnRH-agonist, Decapeptyl®, Ipsen, Belgium) [26], according to local protocol. In the control group, Triptorelin was used mainly when there was a risk to develop ovarian hyperstimulation syndrome (OHSS). In the BC group, GnRHa trigger recently replaced hCG trigger in all patients, regardless of OHSS risk, to maintain low progesterone level during luteal phase [26].

In BC group, a “standard” or “random start” COH was applied depending on the cycle phase, either early follicular or late follicular/luteal phase, respectively [27]. In standard protocol, letrozole 5 mg/day (Femara®, Novartis, Switzerland) was started on cycle day 2, and gonadotropins were administered the following day. GnRH antagonists were initiated after 5 days. In the “random start” protocol, letrozole, gonadotropins, and GnRH antagonist were often administered together during the stimulation. Letrozole administration was discontinued on the ovulation trigger day in both protocols. Follicular development was monitored by pelvic ultrasound scans and serum endocrine profile (luteinizing hormone (LH), estradiol, and progesterone) and ovulation was triggered as soon as at least 2 follicles reached 19–20 mm [10].

In the control group, patients underwent a conventional antagonist COH, without letrozole. Ovulation was triggered according to usual practice, as soon as at least 3 follicles reached 17–18 mm. Oocyte pick-up (OPU) was performed 34-36 h after ovulation triggering. Follicles were aspirated via a single-lumen needle (Cook®, Australia) using transvaginal ultrasound transducer, with an aspiration pressure of 120 mmHg (Cook®, Australia). Each follicle was aspirated individually into a 5 ml Falcon tube and systematically separated from the flush medium.

### Follicular fluid (FF) analysis

After removal of the cumulus-oocyte-complex (COC), each FF was recovered separately and centrifuged at 3000 RPM for 10 min. Only individual samples of > 2 ml enclosing a mature oocyte, without visible contamination of flushing media or blood were stored at − 20 °C for further analysis.

Estradiol (E2), and progesterone (P) were assayed by electrochemiluminescence immunoassay using a competitive immunoassay (Modular E170 – Roche diagnostics, Mannheim, Germany). The inter-assay coefficient of variation was less than 5% for both assays. Testosterone (T) levels were measured by radioimmunoassay (DIA source, Louvain-La-Neuve, Belgium). The inter-assay coefficient of variation was less than 7%. Samples were diluted (1:1000) in Multi assay diluent (MA) for E2 and in the E2-P diluent for P. T was diluted (1:2) in study group only, in fetal calf steroid-free serum.

### Cumulus cell (CC) collection and oocyte handling

Collected COC were washed and incubated individually in Fertilization medium (Cook Medical, USA) under mineral oil for 1 h before denudation. Denudation was performed in 30 μl droplets of Gamete Buffer (Cook Medical, USA) containing 80 IU hyaluronidase (HYASE, Vitrolife, Sweden) for 30 s, and then washed in two 30 μl droplets of enzyme-free Gamete Buffer.

Whenever a BC patient had a male partner, ICSI was performed on a variable proportion of mature oocytes (MII), according to patient’s decision. Embryos obtained were vitrified at 2PN or cleavage stage. If the patient was single, all MII oocytes were directly vitrified after denudation.

In the control group, all MII oocytes were subjected to ICSI. Embryo transfer was performed on day 3 or 5 of culture and the remaining good quality embryos were vitrified according to local protocol. Oocytes and embryos were handled individually in both groups to allow CC per oocyte analysis.

### RNA extraction, reverse transcription (RT) and real-time PCR

For each individual mature oocyte, CC samples were collected separately and centrifuged twice for 10 min (2000 RPM at 4 °C in PBS) to remove culture media and mineral oil.

Total RNA extraction was performed immediately, using RNAqueous®-Micro Total RNA Isolation kit (Thermo Fisher Scientific, USA) according to manufacturer’s instructions. Samples were treated with recombinant DNase I RNase-free (Thermo Fisher Scientific, USA) to remove any potential genomic DNA contamination. RNA was assessed for quantity and purity by spectrophotometry (NanoDrop 2000) and stored at − 80 °C until RT. RT and qPCR were conducted on CC samples with an mRNA concentration > 5 ng/μl. Reverse transcripts of total RNA were prepared using a High Capacity cDNA Reverse Transcription Kit (Thermo Fisher Scientific, USA) with a final reaction volume of 20 μl. Negative controls were performed by replacing the enzyme with water. cDNA samples were stored at − 20 °C until qRT-PCR. *HAS2*, *PTGS2,* and *GREM1* were selected as target genes. Ribosomal protein L19 (*RPL19)* and Hypoxanthine phosphoribosyltransferase-1 (*HPRT1)* were selected as housekeeping genes (validated by Genorm software). Primers were designed using the free Primer3Plus software except for *GREM1* (commercial assay, SigmaAldrich, Austria) (Additional file 1: Table S1). Amplification efficiency ranged between 90 and 110% for each primer pair. Specificity of single PCR products was confirmed by gel electrophoresis for all genes. qPCR experiments were performed on a 7500 Cycler (Applied Biosystems). The reaction mixture contained 5 μl cDNA (1 ng), 200 nM of each primer and 10 μl PowerSYBR® Green PCR Master Mix (Thermo Fisher Scientific, USA) in a final reaction volume of 20 μl. After activation and denaturation (20 s at 50 °C and 10 min at 95 °C), the cDNA was subjected to 40 amplification cycles (15 s at 95 °C and 1 min at 60 °C). All samples were run in triplicate and a No Template Control (NTC) was included for each gene. Gene expression levels were normalized to the geometric mean of the housekeeping genes and fold increases were calculated using the 2^∆∆CT^ method.

### Statistics

Statistical analyses were performed using SPSS 23 (IBM, Brussels, Belgium) on Mac OS X. Mann-Whitney or Student’s t-tests were performed where appropriate. Considering some patients in the study group underwent 2 cycles of Let-COH, and the number of samples per patient was variable in both groups, we performed a Two-way ANOVA with group as a fixed factor and patients as a nested within group random factor to test statistical dependencies of samples using the NCSS 10 Statistical Software 2015 LLC. Kaysville, Utah, USA, ncss.com/software/ncss). All tests were two-tailed and a *P*-value of less than 0.05 was considered statistically significant.

## Results

Biomarkers data were analyzed separately for hCG and GnRHa triggers, as follicular microenvironment differs according to ovulation trigger method [28–30]. Patient and cycle characteristics are shown in Table 1.

**Table 1:** Patients and cycle characteristics

	Study group	Control Group	*P* value
N patients	23	24	
N cycles	27	24	
Age (years)	30.4 ± 3.8	30.8 ± 3.9	0.71
AMH (ng/dl)	2.7 ± 2.1	3.1 ± 1.7	0.32
N stimulation days	9.9 ± 2.6	9.7 ± 1.3	0.8
Total dose of FSH used	2280 ± 1027	1629 ± 624	0.004
E2 level at triggering (pg/ml)	315 ± 225	2144 ± 915	< 0.001
Triggering methods (cycles)			0.22
hCG	9	12	
GnRHa	18	12	
Number of oocytes collected	9.5 ± 5.1	10.9 ± 6.8	0.4
Maturation rate	0.83 ± 0.19	0.87 ± 0.14	0.34

### FF endocrine profile assessment

We assessed the effect of letrozole on steroid levels in the FF to indirectly evaluate its impact on oocyte competence. To avoid potential bias related to differences in follicular size, only FFs of similar volumes were analyzed in both groups (3.4 ± 1.4 vs 3.2 ± 1 ml per follicle, *p* = 0.533). Additionally, after exclusion of contaminated samples, a total of 73 FF samples from 18 control patients, and 66 FF samples from 16 BC patients performing 18 and 20 cycles respectively, were eligible for the FF analysis. First, we confirmed that no difference was observed in FF steroid levels between “standard” and “random start” protocols in the study group, in both ovulation trigger methods (unpublished data). Steroid levels were then compared between study and control groups.

**Fig. 1:**
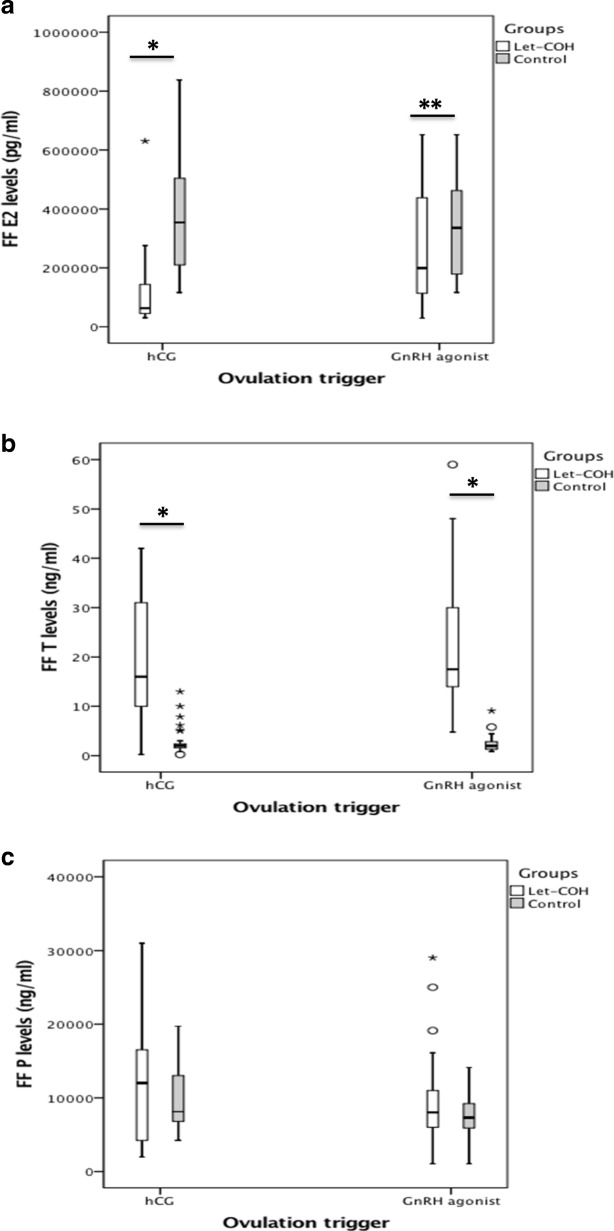
Steroid levels in follicular fluid: estradiol (**a**), testosterone (**b**) and progesterone (**c**) concentrations in study and control groups. Boxplots represent the median, 25th, and 75th percentiles. The whiskers represent 1.5 times the interquartile range, and outliers are identified by circles (out-values) and stars (extreme values). *: *p* < 0.001; **: *p* = 0.011

Estradiol levels were significantly lower in the study compared to the control group, while testosterone levels were significantly higher (Fig. 1a, b). Progesterone levels were comparable between groups (Fig. 1c). Using a nested two-way ANOVA analysis, we confirmed there was no group effect regarding FF volume. However, we observed a significant effect of both patient and group factors for hormonal levels (*p* < 0.001). Interestingly, estradiol levels increased significantly after GnRHa trigger compared to hCG trigger in the study group (median = 194.5 (95.4–438) vs 64.4 (43.8–152.4) ng/ml, respectively, *p* < 0.001) but not in the control group (median = 335.5 (177.5–466.7) vs 354 (179–511) ng/ml, respectively) (Fig. 1a).

### CC gene expression related to oocyte competence

A total of 19 controls (82 CC samples) and 22 BC patients (89 CC samples) performing 19 and 24 cycles respectively, were eligible for the CC analysis. To validate the analysis, we first confirmed that the expression of genes was lower in CC from unfertilized oocytes or low quality embryos compared to CC from mature oocytes resulting in top quality embryos in our control group. However, the difference reached significance only for *HAS2* and *PTGS2* after GnRHa trigger (Additional file 2: Figure S1).

**Fig. 2:**
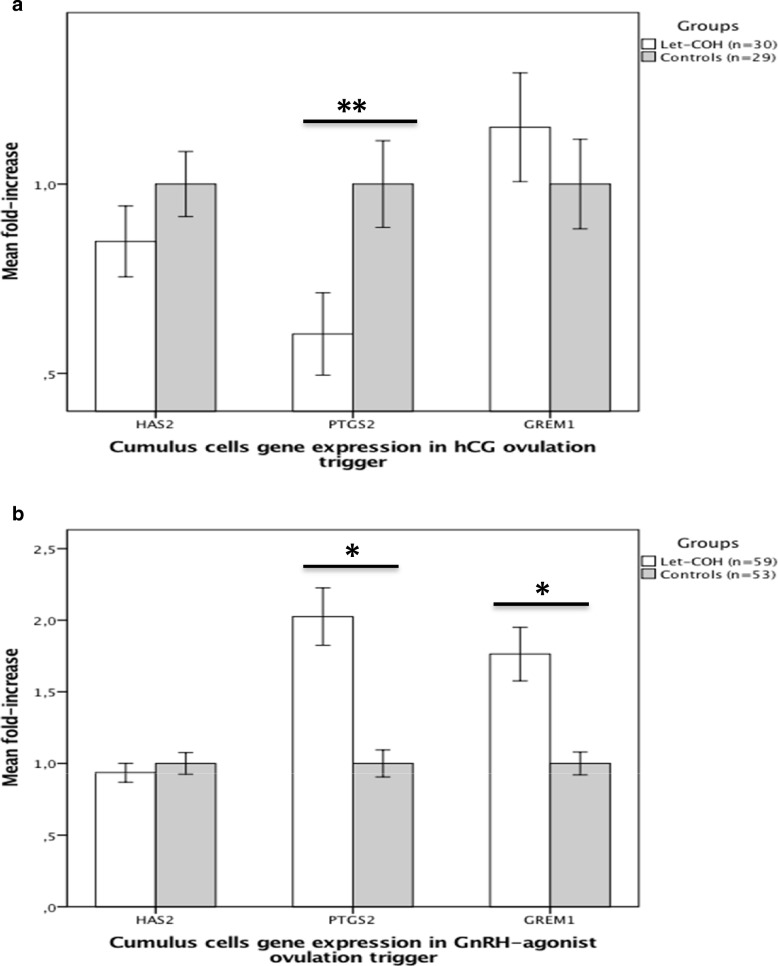
Fold-change in gene expression in cumulus cells in study and control groups after hCG (**a**) and GnRHa (**b**) ovulation trigger, respectively. Results are presented in mean +/− SEM. *: *p* < 0.001; **: *p* = 0.015. HAS2: hyaluronan synthase 2; PTGS2: prostaglandin endoperoxide synthase 2; GREM1: gremlin 1

In the hCG-trigger, expression of *HAS2* and *PTGS2* was lower in the study group (*n* = 8 Let-COH) compared to the control group (*n* = 10 COH), but the difference reached statistical significance only for *PTGS2* (*p* = 0.015) (Fig. 2a). Conversely, when GnRHa was used as the trigger, *HAS2* expression was comparable between groups (*n* = 16 Let-COH and 9 COH), but expression of *PTGS2* and *GREM1* was significantly higher in the study compared to the control group (*p* < 0.001 for both genes) (Fig. 2b). As for FF hormonal levels, nested Two-way ANOVA analysis showed an effect of both patient and group factors (*p* < 0.05).

## Discussion

While hundreds of BC patients have cryopreserved eggs using Let-COH, to date, only two studies have reported specific outcomes of nearly 100 frozen embryo transfers. Success rates were similar to that of the general infertile population after frozen embryo transfer, with implantation and live birth rates per transfer reaching 39.7 to 40.7% and 32.3 to 45%, respectively [18, 31]. A recent study on infertile patients undergoing IVF confirmed the similar cumulative pregnancy rates between Let-COH and conventional COH groups (58.3% vs 65.2% respectively) [19]. Notwithstanding these promising results, oocyte quality has been poorly investigated so far in BC patients undergoing Let-COH for FP. Our study aimed to compare indirect markers potentially related to oocyte quality, following Let-COH in BC patients and conventional COH in infertile patients. Ovulation was triggered with either hCG or GnRHa in both groups, and considering the impact of ovulation triggering on the microenvironment surrounding the oocyte [32], we compared results between groups according to final oocyte maturation trigger methods. In the control group, we observed stable FF steroid levels regardless of ovulation trigger method. Conversely, in the study group triggered with GnRHa, E2 levels increased significantly as compared to hCG trigger, although T levels remained comparably high. Similarly, results of CC gene expression revealed a less favorable profile in the study compared to the control group triggered with hCG, although only difference in *PTGS2* expression reached statistical significance. On the contrary, the expression of *PTGS2* and *GREM1* was significantly improved in the study compared to the control group when triggered with GnRHa. Decreased estradiol/testosterone ratio in Let-COH triggered with hCG may actually generate a suboptimal FF environment. This observation is in accordance with previous studies on infertile patients, in which aromatase inhibitors were used for androgen priming before standard COH to increase follicular sensitivity to FSH. In these studies, high FF testosterone level was associated with reduced oocyte fertilization rates but good embryo quality [33, 34]. The authors suggested a dual effect of androgens on granulosa cell function, as they increase FSH receptors and stimulate steroidogenesis in small antral follicles but may be detrimental in later stages of follicular development. Similarly, in a mouse model, addition of a high dose of an aromatase inhibitor during follicular culture improved maturation rates compared with controls However the fertilization rates were decreased (45% vs 76%) while blastocyst/2-cell embryo ratios were similar between groups [35].

In our study, increased E2 levels following GnRHa trigger in the Let-COH group induced a high *PTGS2* expression, which may be beneficial in terms of oocyte quality. Indeed, estradiol has been previously shown in aromatase knockout mice to be mandatory for PTGS2 induction and ovulation [36]. We hence hypothesize that the FSH surge induced by agonist trigger at the time of letrozole interruption may stimulate aromatase activity to increase estradiol production in the pre-ovulatory follicle. This rise in estradiol level may positively influence oocyte maturity and quality, regardless of follicular testosterone level [37].

Our results are also in accordance with a recent study that showed comparable pregnancy rates in normal responder infertile patients undergoing Let-COH or conventional COH. The authors observed significantly lower E2 and significantly higher T levels in the FF [38].

Our study has several limitations. Follicular size limit determined for ovulation trigger was different between groups. Indeed, more than a decade ago, Oktay et al. showed lower maturation rates when Let-COH was triggered as soon as follicles reached 17-18 mm as in conventional COH. This observation led to a timing modification of ovulation triggering in Let-COH and maturation rate improvement [10]. In our study however, maturation rates were comparable between groups regardless of ovulation trigger, and only FFs of similar size were analyzed to avoid possible bias related to follicular size.

FF steroid concentrations should be interpreted with caution despite the significant difference observed between groups, as high variability of FF steroid concentrations within and between subjects were previously reported; testosterone and progesterone showed higher inter-subject variability while estradiol showed higher intra-subject variability, suggesting that estradiol may be a better marker to indirectly assess oocyte quality [39]. Comparison of CC gene expression between groups should also be interpreted with caution since they remain merely indirect markers of oocyte competence. Moreover, the use of predictive gene panel of oocyte quality expressed in CC has not been validated yet in clinical practice. Finally, difference between high and low quality embryos didn’t reach significance in the hCG triggered control cohort. However, as all our BC patients are now triggered with GnRHa, CC gene expression analysis in the latter was more clinically relevant.

In conclusion, clinical results of Let-COH efficiency in terms of oocytes quality and pregnancy outcomes in BC patients are still limited, and large data will probably be available in several years. We evaluated for the first time Let-COH impact on oocyte microenvironment in BC patients. Our results suggest that GnRHa-trigger may improve oocyte quality in this population.

## Additional files

Additional file 1: **Table S1.** Primer sequences for housekeeping and target genes. (DOCX 14 kb)

Additional file 2: **Figure S1.** Fold-change in gene expression in cumulus cells of good quality embryos compared with poor quality embryos and unfertilized oocytes in the control group, after hCG (a) and GnRHa (b) ovulation trigger, respectively. Results are presented in mean +/− SEM. *: *p* = 0.004; **: *p* = 0.036. HAS2: hyaluronan synthase 2; PTGS2: prostaglandin endoperoxide synthase 2; GREM1: gremlin 1. (DOCX 64 kb)

## Abbreviations

AMH: Anti-müllerian hormone
BC: Breast cancer
CC: Cumulus cells
COC: Cumulus-oocyte-complex
COH: Controlled ovarian hyperstimulation
E2: Estradiol
FF: Follicular fluid
FP: Fertility preservation
FSH: Follicular stimulating hormone
GnRH: Gonadotropin releasing hormone
GREM1: Gremlin1
HAS2: Hyaluronic acid synthase 2
hCG: Human chorionic gonadotropin
HPRT-1: Hypoxanthine phosphoribosyltransferase-1
ICSI: Intracytoplasmic sperm injection
Let-COH: Letrozole associated controlled ovarian hyperstimulation
LH: Luteinizing hormone
MII: Metaphase 2 oocyte
OPU: Oocyte pick-up
P: Progesterone
PCOS: Polycystic ovary syndrome
PN: Pronuclei
PTGS2: Prostaglandin-endoperoxide synthase-2
RPL-19: Ribosomal protein L19
T: Testosterone
