# Supplementary material for: Letrozole-associated controlled ovarian hyperstimulation in breast cancer patients versus conventional controlled ovarian hyperstimulation in infertile patients: assessment of oocyte quality related biomarkers
Source: Reprod Biol Endocrinol. 2019 Jan 3;17:3. doi: 10.1186/s12958-018-0443-x (PMC6318989; doi:10.1186/s12958-018-0443-x)
Supplement: Supplementary file 1 — Table S1. Primer sequences for housekeeping and target genes. (DOCX 14 kb) [file 12958_2018_443_MOESM1_ESM.docx]

Additional file 1: Table S1: Primer sequences for housekeeping and target genes.

| Gene  Symbol | Forward Primer | Reverse primer | Accession N° | Amplicon length |
| --- | --- | --- | --- | --- |
| RPL19 | TGAAATCGCCAATGCCAACT | TTCCGCTTACCTATGCCCAT | NM_000981.3 | 158 |
| HPRT1 | CCTGGCGTCGTGATTAGTGAT | GAGCACACAGAGGGCTACAA | NM_000194.2 | 189 |
| HAS2 | TCCCGGTGAGACAGATGAGT | TTAAAATCTGGACATCTCCCCCA | NM_005328.2 | 269 |
| PTGS2 | CTGTTGCGGAGAAAGGAGTC | TCATGGAAGATGCATTGGAA | NM_000963.3 | 261 |
| GREM1 | AATGAGATTGCCAGAAAGTG | GAGGAGTTGGTTTGGTTTAG | NM_013372.6 | 132 |

RPL19: ribosomal protein L19; HPRT: hypoxanthine phosphoribosyl transferase 1; HAS2: hyaluronan synthase 2; PTGS2: prostaglandin endoperoxide synthase 2; GREM1: gremlin 1
