# Supplementary figures and images for: Letrozole-associated controlled ovarian hyperstimulation in breast cancer patients versus conventional controlled ovarian hyperstimulation in infertile patients: assessment of oocyte quality related biomarkers
Source: Reprod Biol Endocrinol. 2019 Jan 3;17:3. doi: 10.1186/s12958-018-0443-x (PMC6318989; doi:10.1186/s12958-018-0443-x)

Additional file 2: Figure S1a


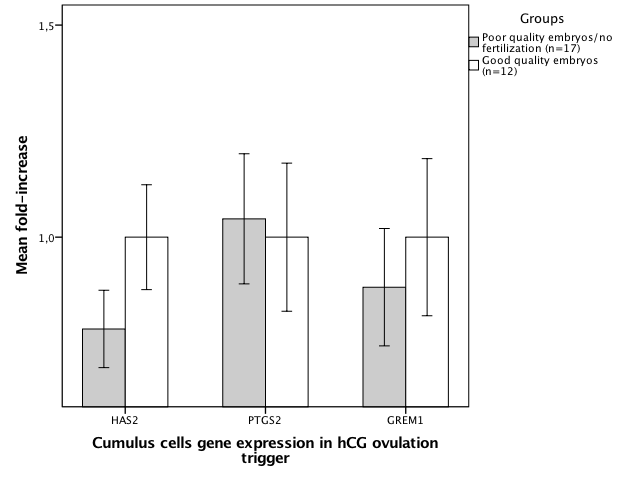


Figure S1b


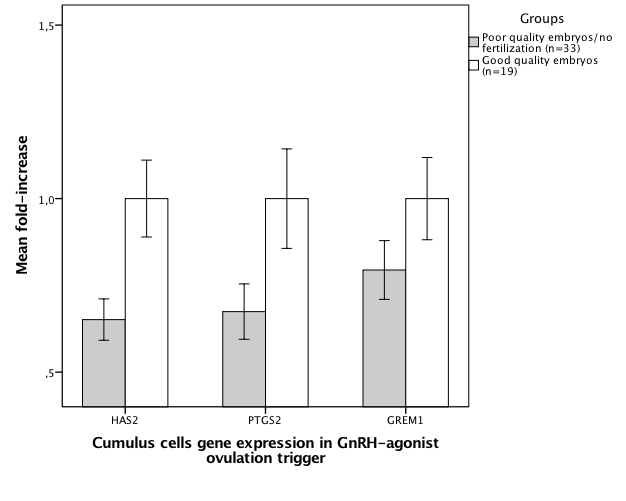


*****

******

Supplement: Supplementary file 2 — Figure S1. Fold-change in gene expression in cumulus cells of good quality embryos compared with poor quality embryos and unfertilized oocytes in the control group, after hCG (a) and GnRHa (b) ovulation trigger, respectively. Results are presented in mean +/− SEM. *: p = 0.004; **: p = 0.036. HAS2: hyaluronan synthase 2; PTGS2: prostaglandin endoperoxide synthase 2; GREM1: gremlin 1. (DOCX 64 kb) [file 12958_2018_443_MOESM2_ESM.docx]
